# The Suspected Infected Prosthetic Joint: Clinical Acumen and Added Value of Laboratory Investigations

**DOI:** 10.1371/journal.pone.0131609

**Published:** 2015-07-16

**Authors:** Cathy A. Petti, Gregory J. Stoddard, Merle A. Sande, Matthew H. Samore, Keith E. Simmon, Aaron Hofmann

**Affiliations:** 1 Department of Medicine, University of Utah Hospital and Clinics, Salt Lake City, Utah, United States of America; 2 Associated Regional and University Pathologists Laboratories Institute of Clinical and Experimental Pathology, Salt Lake City, Utah, United States of America; 3 Department of Orthopaedic Surgery, University of Utah Hospital and Clinics, Salt Lake City, Utah, United States of America; University Hospital of the Albert-Ludwigs-University Freiburg, GERMANY

## Abstract

Consensus definitions have emerged for the discrimination between infected and uninfected prosthetic joints but diagnostic uncertainty often occurs. We examined the accuracy of orthopaedic surgeons’ assessments to diagnose the infected prosthetic hip or knee and elucidated the added value of laboratory parameters. A prospective cohort study of patients undergoing revision arthroplasty of hip or knee was conducted over a one-year period. Orthopaedic surgeons’ determinations prior to arthroplasty were recorded. A reference diagnostic standard was determined retrospectively by independent review from 3 infectious diseases physicians. Patients were followed up to 12 months. For 198 patients enrolled, 228 surgical encounters (110 knee, 118 hip) were classified by independent reviewers as 176 uninfected and 52 infected. Orthopaedic surgeons’ preoperative diagnoses of infection had high diagnostic accuracy (sensitivity 89%, specificity 99%, PPV 98%, NPV 97%). Addition of intraoperative findings and histopathology improved their diagnostic accuracy. Addition of culture and PCR results improved sensitivity of diagnostic determinations but not specificity. We provide evidence that clinical acumen has high diagnostic accuracy using routine preoperative parameters. Histopathology from intraoperative specimens would improve surgeons’ diagnostic accuracy but culture and PCR from intraoperative specimens could create greater diagnostic uncertainty. This study is critical to further our understanding of the added value, if any, of laboratory testing to support clinical decision making for the suspected infected joint and allow us to identify diagnostic gaps for emerging technologies to fill that will improve our ability to diagnose the infected prosthetic joint.

## Introduction

Osteoarthritis (degenerative arthritis) is commonly considered a disease of aging and the leading indication for total joint replacement surgery of the hip and knee. According to the National Hospital Discharge Survey (NHDS), 332,000 total hip replacements and 719,000 total knee replacements were performed in the United States in 2010, with older adults aged ≥ 65 years comprising 50% and 53%, respectively [1]. In the context of a growing aging population, it is estimated that by 2030, a total of 4 million arthroplasties will be performed each year in the US [2]. Interestingly, recent NHDS data suggest that the *percentage increase* of hip replacements is now greater among younger age groups (45–64 years) and decreasing among older age groups [3]. Aseptic loosening is the most frequent complication following total joint replacement, but the second and more devastating complication is infection of the orthopedic device (prosthetic joint infection). Infections are commonly caused by bacteria, namely, *Staphylococcus aureus*, coagulase-negative staphylococci, beta-hemolytic streptococci, and gram-negative bacilli. Alarmingly, while the numbers of arthroplasties are increasing steadily, the incidence of prosthetic hip and knee infections have remain unchanged over the last two decades with rates reported to range between 0.5 and 5% [4–7]. Prosthetic joint infections may cause temporary and long-standing impairment in a patient’s functional status and quality of life [8,9], and have considerable economic impact estimating to cost the healthcare system approximately $50,000 per infected episode [10–12].

The diagnosis of the infected joint has challenged primary care physicians, infectious diseases specialists, and orthopaedic surgeons for decades [13–15]. Discrimination between an infected and uninfected prosthetic joint by physicians before or at the time of surgery is critical because their surgical and medical managements differ. Patients with infected joints may undergo surgical debridement and retention of prosthesis, single-stage revision arthroplasty, two-stage revision arthroplasty, amputation, or athrodesis, followed by a prolonged course of antimicrobial therapy. Patients without infected joints may undergo a single-stage revision arthroplasty and no antimicrobial therapy.

Identifying a patient with an infected joint is difficult because patients with infections have varied clinical presentations, and no preoperative or intraoperative investigation alone has sufficient diagnostic accuracy. Observation of a draining sinus tract on physical examination or purulence at the time of surgery is highly specific, but not always present [16]. Measurements of inflammatory markers such as erythrocyte sedimentation rate (ESR) or C-reactive protein (CRP) have high negative predictive values when normal, but they are poorly predictive of infection when elevated [17]. Microbiologic data, a focal point in the decision-making process, often yield false-positive results from contaminating skin microflora[18], and false-negative results from failure to detect adherent biofilm microorganisms or cultures being compromised by prior antimicrobial therapy [19]. Some experts advocate adoption of advanced technologies such as broad-range amplification and sequencing, PCR electrospray-ionization mass spectrometry, microarrays or unique biomarker analyses to improve diagnostic certainty but these methods also have limitations [20–25].

In the absence of a single laboratory test that accurately and reliably discriminates infected from uninfected joints, microbiology and biochemical markers often are seen as barriers to effective care. Interestingly, many test solutions are being offered to improve diagnostic certainty without a rigorous examination of the diagnostic accuracy of current medical decision making practices of the infected joint, or an understanding of laboratory test characteristics that would improve diagnostic certainty. The current standard for diagnosing prosthetic joint infections relies on an orthopaedic surgeon’s ability to synthesize and interpret data from a patient’s clinical history (acute or chronic pain of prosthesis), findings on physical examination (sinus tract or wound drainage), plain films of prosthesis, laboratory test results (CRP, ESR and cultures), and investigations at revision arthroplasty (visual inspection, histopathology of frozen tissue) [5, 26, 27]. We examined surgeon’s diagnostic accuracy based on routinely available data, and used a novel approach by developing an independent reference standard for infection based on the determinations of three infectious diseases physicians. We also sought to elucidate the gaps in our present diagnostic approach and determine the added benefit of various laboratory investigations. Since culture-negative infections are common, we applied a culture-independent method, nucleic acid amplification and sequencing, to improve diagnostic accuracy.

## Methods

### Study Population

All patients ≥ 18 years old undergoing revision hip or knee arthroplasty for either mechanical failure (aseptic loosening) or infection were invited to participate in this prospective study over a twelve month period. Since follow-up interviews on quality of life measures were part of the study design, only individuals able to understand and provide informed consent were eligible to participate. Participants were consented for permission to use their excess specimens collected during normal surgical procedures for additional testing and post-op follow-up interviews. Of 206 patients eligible to participate in the study, 198 enrolled with informed consent, 5 could not be contacted, and 3 declined participation. The University of Utah institutional review board approved this prospective study (#13329) including the consent procedure, and before enrollment, written informed consent was obtained from all patients and documented in a secure record.

### Study design

Clinical data were collected from the hospital computer-based medical record. Orthopaedic clinic notes prior to revision arthroplasty were used to ascertain the orthopaedists’ diagnostic determination; diagnoses were classified as uninfected (aseptic loosening or reimplantation), infected, or uncertain (rule out infection). Orthopaedic surgeons who specialize in hip and knee arthroplasty were used (n = 3). Three independent infectious diseases specialists retrospectively reviewed clinical records with pre-, intra- and postoperative data to classify each surgical encounter as uninfected (aseptic loosening or reimplantation) or infected. Patient telephone interviews and review of hospital medical records were conducted to ascertain clinical outcomes up to 12 months following hospital discharge.

At the time of surgery at least 3 periprosthetic tissue or synovial fluid samples were collected from all patients and processed for Gram stain, aerobic and anaerobic cultures by standard laboratory protocols. Histologic examination was performed for each patient on frozen and permanent tissue sections. Following routine culture, remaining tissue and/or synovial fluid from each sample was frozen at -70°C. DNA extraction, amplification and sequencing of the 16S rRNA gene from intraoperative material were performed by standard laboratory protocols. Specifically, DNA was extracted in batches of 8–12 samples in a biosafety cabinet with a negative extraction control using 200 μl of PCR water (MO BIO Laboratories, Carlsbad, CA) for each batch. DNA was extracted with either QIAamp DNA Blood Mini kit (Qiagen Inc. Valencia, CA) or MasterPure DNA Purification Kit (Epicenter Biotechnologies, Madison, WI) according to the manufacturer’s instructions. PCR was performed in 20 μl volume containing 1X Taq buffer; 0.25 U of TaKaRa Taq; 2.0mM MgCl_2_, 200 μM dNTP (Takara Bio, Inc., Shiga, Japan); 0.2 μM each primer; and 2 μl of template. Before template was added, master mix was filtered through a 100-kDa filter (Millipore, Billerica, Mass.) to reduce possible contaminating DNA. The primers for amplification of the 16S rRNA gene were 534F (5’-GTGCCAGCAGCCGCGGTA-3’) and 1194R (5’-ACGTCATCCCCACCTTCCTC-3’). Each run included a negative PCR control using 2 μl of PCR water as the template. The PCR mixtures were amplified using touchdown PCR procedure which had an initial hold at 94°C for 5 min and then 50 cycles of denaturing at 94°C for 30s, annealing for 30s at 70°C, and extension at 72°C for 2 min. The annealing temperature was lowered 1°C per cycle to a final temperature of 50°C after 20 cycles. The reaction ended with a final extension at 72°C for 2 min and a hold at 4°C. PCR reaction and amplicon size were confirmed by gel electrophoresis.

Cloning of PCR products was performed using TOPO TA Cloning kit (Invitrogen Corp. Carlsbad, CA) with pCR2.1-TOPO vector according to the manufacturer’s instructions. Negative extraction and negative PCR controls with each batch of samples were also cloned regardless if a visible band was present to define population of background microbial DNA. Transformation was done into chemically competent TOP10 cells according to the instruction provided and plated on LB kanamycin (50 μg/ml) plates (Teknova Inc., Hollister, CA) supplemented with X-gal (Invitrogen). After incubation, 8 colonies for each sample were inoculated into 2 ml 96-deep well plates (VWR International) containing 400 μl of LB kanamycin (50 μg/ml) broth. Following a second incubation, clones were amplified by direct inoculation of 1 μl of overnight culture into PCR reaction performed the same as above with the exception of different primers: M13F-20 (5’-GTAAAACGACGGCCAG-3’) and M13R (5’-CAGGAAACAGCTATGAC-3’). The PCR reactions were amplified using PCR procedure which had an initial hold at 94°C for 5 min and then 25 cycles of denaturing at 94°C for 30s, annealing for 30s at 55°C, and extension at 72°C for 2 min. The reaction ended with a final extension at 72°C for 2 min and a hold at 4°C. PCR reactions and amplicon size were confirmed by gel electrophoresis. Samples were then sent to Agencourt Biosciences for purification and sequencing. Sequencing was performed in one direction using 1194R primer. ABI files were provided by Agencourt Biosciences and were manually edited in Contig Express (Vector NTI, Invitrogen). Only clones generating 400bp of readable sequence were analyzed further. Sequences were compared to related reference sequences with the SmartGene database (version 3.2.3r8).

### Definitions

A prosthetic joint was defined as uninfected or infected based on the consensus of independent reviews from three infectious diseases specialists. While many guidelines have been published to help classify prosthetic joints as infected or uninfected [5, 26, 27], no gold standard exists for discriminating between the infected and uninfected joint. We used the diagnostic determinations of infectious diseases specialists as the closest approximation, which we refer to as the reference standard. Infected patients were classified into early infection (≤ 4 weeks from arthroplasty) or late infection. Wound drainage was defined as any persistent serous, serosanguinous, or purulent fluid draining from the surgical site. In the absence of a gold standard, an abnormal result for joint fluid was defined as >10,000 white blood cells per microliter, positive culture, or both. Histology was classified as acute inflammation (> 5 neutrophils per high-power field); chronic inflammation; acute and chronic inflammation; or no inflammation.

### Statistical Analysis

Comparisons of patient characteristics between patients with infected and uninfected prosthetic joints were made using the chi-square test or Fisher’s exact test, as appropriate, for categorical variables. For continuous variables, an independent samples t test was used. Inter-rater agreement of the dichotomous infection classification among the three infectious disease reviewers was assessed with the kappa coefficient. The *p* values for the reported odds ratios were generated using a chi-square test or Fisher’s exact test for the 2 × 2 tables used in computing the test characteristics. If a zero cell count was observed in the 2 × 2 table, the odds ratio and confidence interval were computed after adding 0.5 to each cell of the table [28, 29].

## Results

For 198 patients enrolled, 228 surgical encounters (110 knee, 118 hip) were classified by independent reviewers as 176 uninfected (152 aseptic loosening, 24 reimplantations) and 52 infected (15 early, 37 late infections). Patient demographics are provided in Table 1.

**Table 1:** Patient characteristics.

	Infected [n = 52]	Not Infected [n = 176]	P Value
**Female gender, n (%)**	22 (42)	65 (37)	0.48
**Age, mean±SD, years (Min-max)**	67±14 (30–89)	66±12 (26–89)	0.82
**Diabetes mellitus, n (%)**	7 (13)	29 (16)	0.60
**Rheumatoid arthritis, n (%)**	5 (10)	17 (10)	0.99
**Surgical site, n (%)**			
Hip	34 (65)	84 (48)	0.025
Knee	18 (35)	92 (52)	
**Renal insufficiency, n (%)**	7 (15)	11 (7)	0.08
**Immunosuppression, n (%)**	7 (13)	15 (9)	0.29
**Malignancy, n (%)**	4 (8)	9 (5)	0.50
**Attending Surgeon, n (%)**			
Surgeon A	96 (55)	28 (54)	>0.99
Surgeon B	57 (32)	12 (33)	
Surgeon C	23 (13)	7 (13)	

### Inter-rater agreement

The agreement of classifications, infected versus uninfected, by three infectious diseases reviewers was examined. A high degree of inter-rater consistency was observed, κ 0.93 (95% confidence interval (CI), 0.89 to 0.98).

### Preoperative clinical predictors

Table 2 summarizes preoperative clinical parameters that were available to orthopaedic surgeons for clinical assessment.

**Table 2:** Analysis of pre-operative variables (n = 228).

	Infected [n = 52] Number (%)	Not Infected [n = 176] Number (%)	Odds Ratio % (95% CI), P value	Sensitivity % (95% CI)	Specificity % (95% CI)	Positive Predictive Value % (95% CI)	Negative Predictive Value % (95% CI)
Patient age ≥ 65 years	28 (21)	105 (79)	0.8 (0.4–1.5), P = 0.46	54 (40–68)	40 (33–48)	21 (15–29)	75 (65–83)
Hip surgical site relative to knee surgical site	34 (29)	84 (71)	2.1(1.1–3.9), P = 0.025	65 (51–78)	52(45–60)	29 (21–38)	84 (75–90)
Joint age 0–18 months relative to >18 months	44 (42)	62 (58)	10 (4–22), P<0.001	85 (72–93)	64 (56–71)	42 (32–52)	93 (87–97)
Prior revision arthroplasty of same joint	15 (16)	76 (84)	0.53 (0.27–1.04), P = 0.064	29 (17–43)	57 (49–64)	17 (10–26)	73 (65–80)
Prior prosthetic infection of same joint	29 (45)	35 (55)	5.1 (2.6–9.8), P<0.001	56 (41–70)	80 (73–86)	45 (33–58)	86 (80–91)
Pain	30 (18)	138 (82)	0.4 (0.2–0.7), P = 0.003	58 (43–71)	22 (16–28)	18 (12–24)	63 (50–75)
Wound drainage	35 (95)	2 (5)	179 (40–810), P<0.001	67 (53–80)	99 (96–100)	95(82–99)	91 (86–95)
Knee surgical site subgroup: effusion present	13 (28)	34 (72)	4.4 (1.5–14), P = 0.006	72 (47–90)	63 (52–73)	28 (16–43)	92 (82–97)
CRP abnormal	41 (52)	38 (48)	61 (14–264), P<0.001	95 (84–99)	75 (67–82)	52 (40–63)	98 (94–100)
ESR abnormal	35 (52)	32 (48)	33 (11–101), P<0.001	90 (76–97)	79 (72–85)	52 (40–65)	97 (92–99)
Radiographic evidence of implant loosening	8 (9)	83 (91)	0.30 (0.1–0.7), P = 0.004	23 (10–40)	50 (43–58)	9 (4–17)	76 (67–83)
Abnormal fluid from joint aspiration	11 (92)	1 (8)	126 (10–1550), P<0.001	85 (55–98)	96 (79–100)	92 (62–100)	92 (74–99)
Abnormal fluid from joint aspiration (when wound drainage absent)	5 (83)	1 (17)	172 (6–4827), P<0.001	100 (48–100)	96 (79–100)	83 (36–100)	100 (85–100)
Preoperative diagnosis, Infected relative to not infected	40 (98)	1 (2)	1328 (151–11685), P<0.001	89 (76–96)	99 (97–100)	98 (87–100)	97 (93–99)
Preoperative diagnosis, Possibly infected relative to not infected	7 (44)	9 (56)	5 (3–10), P<0.001	58 (28–85)	95 (91–98)	44 (20–70)	97 (93–99)

Summation may not equal sample size because tests not ordered.

CI = confidence interval

Hip prostheses were twice as likely to be infected compared with knee prostheses (odds ratio, 2.1; 95% CI 1.1 to 3.9; P<0.025). Patient age, including those greater than 65 years, had no affect on likelihood of being infected at the time of revision arthroplasty. Patients with prosthetic joints for 18 or less months were ten times more likely to be infected compared with patients with implants >18 months. A history of prior revision arthroplasty for any indication (e.g., trauma, aseptic loosening, infection) of the same joint did not increase the likelihood of infection whereas prior prosthetic infection of same joint was associated with a five-fold increase (odds ratio 5.1; 95% CI 2.6 to 9.8; P<0.001). Pain, a common presenting symptom, was more common in uninfected patients and appeared to be a surrogate marker for patients with radiographic evidence of implant loosening. For findings on physical examination, wound drainage was an excellent predictor of infection (positive predictive value 95%, negative predictive value 91%), but lacked sensitivity. When limited to patients with knee arthroplasties, patients with knee effusion were four times more likely to be infected than those without effusion.

### Preoperative diagnostic predictors

Patients with elevated CRP and ESR values were more likely to be infected at revision arthroplasty, with normal levels highly predictive of no infection. Radiographic evidence of implant loosening tended to decrease the likelihood of infection. Joint aspirations prior to revision arthroplasty were performed in only 40 (14 infected, 26 uninfected) of 228 surgical encounters. Abnormal synovial fluid was an extremely useful predictor of infection (odds ratio 126; 95% CI 10 to1550; P<0.001) (Table 2). In the absence of wound drainage, all test characteristics of joint aspirations improved except for its positive predictive value.

### Accuracy of clinical decision-making

The orthopaedic surgeon’s preoperative diagnosis of infection was highly predictive (odds ratio 1328; 95% CI 151 to 11685; P<0.001) of an accurate diagnosis (Table 2). When excluding patients with wound drainage, their diagnostic accuracy still had high positive and negative predictive values at 92% and 98%, respectively (Table 3). In terms of all test characteristics, no single preoperative variable exceeded the performance of physician decision-making in predicting infection. Excluding patients for whom surgeons were uncertain of the diagnosis, orthopaedic surgeons made diagnostic errors in 6 (2.6%) of 228 surgical encounters.

**Table 3:** Analysis of orthopaedic surgeon preoperative diagnosis when wound drainage is absent (n = 191).

	Infected [n = 17] Number (%)	Not Infected [n = 174] Number (%)	Odds Ratio % (95% CI), P value	Sensitivity % (95% CI)	Specificity % (95% CI)	Positive Predictive Value % (95% CI)	Negative Predictive Value % (95% CI)
Orthopaedic surgeon preoperative diagnosis, Infected relative to not infected	11 (92)	1 (8)	451 (46–4386), P<0.001	73 (45–92)	99 (97–100) (97–100)	92 (62–100)	98 (94–99)
Orthopaedic surgeon preoperative diagnosis, Possibly infected relative to not infected	2 (18)	9 (82)	3.01(1.21–7.52) P = 0.045	33(4–78)	95 (90–98)	18 (2–52)	98 (94–99)

For patients whom orthopaedic surgeons could not clearly define infection as an indication for revision arthroplasty, patients were 5 times more likely to be infected relative to those preoperatively diagnosed as uninfected (odds ratio 5; 95% CI 3 to 10; P<0.001). Table 4 delineates the clinical parameters and 12 month outcomes of these 16 patients. Four of 7 infected patients had intraoperative findings suggestive of infection. Eight of 9 uninfected patients did not have intraoperative findings suggestive of infection.

**Table 4:** Clinical Parameters and Outcome of Patients with Uncertain Pre-operative Diagnosis (n = 16).

Diagnostic Determination by Independent Review	Antibiotics Prior to Joint Aspiration and Surgery	Joint Age in Months	Wound Drainage	Abnormal CRP or ESR	AbnormalSynovial Aspirate	Intraoperative Note Consistent with Infection	Abnormal Histopathology	Positive Culture or PCR	Antimicrobial Therapy at Hospital Discharge	Infection at 12 Months
*Uninfected Patients*										
1	No	0.5	No	No	Not done	No	No	No	No	No
2	No	15	No	Yes	No	Yes	Yes(Chronic Inflammation)	No	No	Yes (at 2 months)
3	No	1.75	No	No	Not done	No	Not done	No	No	No
4	No	17	No	Yes	No	No	Not done	No	No	No (at 6 months)
5	No	56	No	No	No	No	No	No	No	Yes (at 1 month)
6	No	8	No	No	No	No	Not done	No	No	Unk
7	No	44	No	No	No	No	Yes (Chronic Inflammation)	No	No	No
8	No	Unk	No	No	No	No	Yes (Chronic Inflammation)	No	No	No
9	Yes	Unk	No	Yes	No	No	Yes (Acute Inflammation)	No	Yes	No
*Infected Patients*										
1	No	0.4	Yes	Not done	No	Yes	Not done	No	Yes	No
2	No	0.6	Yes	Yes	Not done	Yes	Not done	Yes	Yes	Yes
3	No	3.3	No	Yes	Not done	Yes	Yes (Acute Inflammation)	Yes	Yes	No
4	No	0.75	Yes	Yes	Not done	No comment	Not done	Yes	Yes	No
5	No	0.6	Yes	Not done	Not done	Yes	Not done	Yes	Yes	No (at 9 months)
6	No	1	Yes	Not done	Not done	No	Not done	Yes	Yes	No
7	No	6	No	Yes	Not done	No	No	Yes	Yes	No

We evaluated orthopaedic surgeons’ diagnostic accuracy for patients with failing arthroplasty from aseptic loosening that had 12 month follow-up to look for possible missed infection (Table 5). For 228 surgical encounters, 182 (80%) had follow-up after hospital discharge with 165 (72%) having an assessment at a minimum of 12 months post revision arthroplasty. Only 6 (5%) of 120 patients with the preoperative diagnosis of mechanical failure developed a prosthetic joint infection up to 12 months following revision arthroplasty.

**Table 5:** Orthopaedic Surgeon’s Preoperative Diagnosis with Patient Outcome (n = 182).

Diagnostic Determination	Total Number of Surgical Encounters	Number of Encounters with Follow-up ≥ 12 months	Number of Surgical Encounters with Infection (%)
Mechanical	144	120	6 (5%)
Reimplantation	26	19	3 (16%)
Uncertain	16	15	3 (20%)
Infected	42	28	11 (40%)

### Added value of intraoperative investigations

After we examined the diagnostic accuracy of orthopaedic surgeon’s preoperative diagnosis, we then evaluated the potential added value of data collected intraoperatively and postoperatively to improve orthopaedic surgeon’s diagnostic accuracy. Information from visual inspection intraoperatively and histopathological examination from frozen tissue sections would theoretically have changed the diagnosis from uninfected to infected in 7 patients, and infected to uninfected in 0 patients (Table 6). All test characteristics improved except for positive predictive value–this observation can be explained by the presence of acute inflammation on histopathology changing 3 diagnostic determinations from uninfected to infected. For 2 of these 3 patients, the preoperative diagnosis was mechanical failure, patients did not receive antibiotics postoperatively and had no evidence of infection at 12 months. For the third patient, the preoperative diagnosis was reimplantation, patient did not receive antibiotics postoperatively, and returned 6 months later with a failing joint of uncertain etiology.

**Table 6:** Orthopaedic Surgeon’s Diagnostic Determinations (n = 212).

Diagnostic Determination of Infection	Infected [n = 41] Number (%)	Not Infected [n = 171] Number (%)	Odds Ratio % (95% CI), P value	Sensitivity % (95% CI)	Specificity % (95% CI)	Positive Predictive Value % (95% CI)	Negative Predictive Value % (95% CI)
Pre-operative	40 (98)	1 (2)	1328 (151–11685), P<0.001	89 (76–96)	99 (97–100)	98 (87–100)	97 (93–99)
Intra-operative (Visual inspection and histopathology)	44 (92)	4 (8)	1793 (195–16447), P<0.001	98 (88–100)	98 (94–99)	92 (80–98)	99 (97–100)
Post-operative (Culture and PCR result from peri-prosthetic tissue)*	45 (55)	37 (45)	317 (19–5263), P<0.001	100 (92–100)	78 (71–84)	55 (44–66)	100 (97–100)

*Diagnostic accuracy based on assumption that orthopaedic surgeons would rely exclusively on these post-operative parameters.

## Discussion

The study of physician assessments of clinical data is extremely important to better understand physician judgment in routine practice, to provide supporting evidence for consensus guidelines, and to assist in defining the role of adjunctive technologies to improve diagnostic accuracy. Medical misdiagnosis and strategies to reduce or prevent future errors have been studied in clinical syndromes such as appendicitis, pulmonary embolism, and malignancy, and have found that incorrect diagnoses result from physician cognitive bias, laboratory error, or misinterpretation of test results [30, 31]. For the infected joint, consensus and expert opinion deem a physician’s summation and interpretation of available clinical and laboratory data as the gold standard but to our knowledge, this study is the first to rigorously evaluate this clinical practice. Our audit of orthopaedic surgeons’ decision-making for the failing hip or knee arthroplasty prior to surgery demonstrated their high diagnostic accuracy, and the audit demonstrated that laboratory investigations did not unambiguously add value and may contribute to diagnostic uncertainty.

Similar to findings from prior studies [14, 15,17, 32–38], we found no preoperative and intraoperative variable that would consistently and reliably predict infection. Normal CRP and ESR were helpful to exclude infection, but when elevated, had poor positive predictive values. Although the presence of wound drainage was an excellent predictor of infection, its sensitivity was only 67%. In terms of all test characteristics (sensitivity, specificity, positive predictive value, negative predictive value), no preoperative variable was better than orthopaedists’ clinical acumen to diagnose the infected joint even when wound drainage was absent. Our analysis also showed that had orthopaedic surgeons relied on intraoperative observations of purulence or necrotic tissue and results from histopathology, their diagnostic accuracy would have improved. These findings suggest that the value of intraoperative observations should not be underestimated and that histopathology be incorporated as standard practice for all patients undergoing surgery for a failing arthroplasty regardless of surgical indication.

Orthopaedic surgeons were unable to classify patients as infected or uninfected in 16 patients. Based on the available data, it is unclear why the surgeons were unable to classify the patient pre-operatively. Orthopaedic surgeons made preoperative diagnostic errors in only 2.6% of patients. Five patients were inaccurately classified preoperatively as not being infected. Two of these patients had no wound drainage, but methicillin-resistant *Staphylococcus aureus* recovered from intraoperative cultures; one patient had no wound drainage, negative intraoperative cultures and *S*. *aureus* detected by 16S rRNA PCR from periprosthetic tissue; and two patients (one with wound drainage and one without) had multiple intraoperative cultures and 16S rRNA PCR with coagulase-negative staphylococci. The one patient with negative culture but *S*. *aureus* detected by PCR was not treated with antibiotics and 5 months later developed a prosthetic joint infection with β-hemolytic Group A streptococcus. The independent reviewers diagnosed these 5 patients as being infected with a high degree of inter-rater consistency (for all 5 patients, all 3 reviewers agreed). The remaining one of 6 patients was inaccurately classified preoperatively by orthopaedic surgeons as being infected. The patient had no wound drainage, all 4 intraoperative tissue specimens were negative for bacteria by culture and PCR, and histopathology demonstrated no evidence of acute inflammation. The patient was treated empirically for infection with vancomycin and levofloxacin without recurrence. For this patient, the reviewers did not have complete consensus (2 agreed infection absent; one was uncertain).

Had orthopaedic surgeons relied solely on any positive culture or positive PCR result from periprosthetic tissue/fluid as an indicator of infection (and all negative cultures and negative PCR as an indicator of non-infection), the specificity and positive predictive values of their diagnostic accuracy would have significantly decreased with an increase in sensitivity and negative predictive value. We believe that this observation can be explained by understanding the pathogenesis of infection and pathogen detection methods. The most common causes of prosthetic joint infections are *Staphylococcus aureus* and coagulase-negative staphylococci, two organisms associated with contamination from skin microbiota that may contribute to false-positive results [18, 39]. Culture-negative infections also challenge clinical decisions because a negative culture does not necessarily indicate the absence of infection. When we performed non-culture based methods like broad-range PCR technology in hopes to improve diagnostic certainty, we found that in some cases molecular methods were negative even in the presence of wound drainage and positive cultures. Molecular methods have known limitations such as suboptimal performance from PCR inhibitors, contamination from PCR reagents, human DNA competition, or small sample sizes (microliters) [22]. Hence, reliance on results of culture and/or PCR from intraoperative specimens (two variables generally considered having high diagnostic value when positive) could decrease diagnostic accuracy. Surprisingly, false-negative culture or PCR results did not create diagnostic uncertainty. These observations are important findings because it serves as a guide for requisite performance characteristics of future tests, and how best to apply test results. In the setting of high diagnostic accuracy of experienced orthopaedic surgeons, this study suggests that an innovative diagnostic would require greater specificity to add appreciable value over an orthopaedics surgeon’s clinical acumen using routine pre-operative investigations. And a new diagnostic test as a single intervention to improve accuracy may not necessarily increase diagnostic certainty or serve as a diagnostic error-reduction strategy.

We acknowledge several limitations of this study. The reference standard was based on consensus of infectious diseases specialists from the same institution and classifications potentially could differ among clinicians at other academic or private practice settings. Additionally, our cohort had only 52 infected patients, and with higher numbers of infected patients, our statistical analyses and conclusions could be different. It is important to emphasize that the field of infectious diseases diagnostics is changing rapidly and our understanding of the role of individual and community of microorganisms implicated in infections continue to evolve. With greater research in next generation sequencing and metagenomics, we may find that organisms once classified as “PCR contaminants” may in fact be true pathogens. Conversely, with more advanced technologies, we also may find that culture-based methods have more limitations than those currently known today.

There are multiple, unique strengths of our study. To our knowledge, this study is the first to examine the accuracy of clinical decision-making and establish a diagnostic determination of infection by independent review as a reference standard. Additionally, we applied 16S rRNA gene amplification and sequencing to tissue specimens for additional microbiological certainty since culture-independent methods generally are not compromised by antecedent antimicrobial therapy, poor growth from fastidious microorganisms, or require special media/transport for isolating anaerobes. We acknowledge that the results of this study may not translate to other orthopaedic practices. Three orthopaedic surgeons with at least 15 years of experience were involved in all our surgical cases. While we believe that 12 months follow-up significantly strengthened the validity of our findings, we also acknowledge that prosthetic joint infections can occur greater than 12 months following revision arthroplasty and some patients may have developed recurrent infection beyond our study period. Indeed, our rate of infection following revision arthroplasty was quite high but the patient population was heterogeneous with many having multiple co-morbidities and a history of recurrent infections. Finally, reimplantations represented ~11% of surgical indications which could have increased the diagnostic accuracy of orthopaedic surgeons in this study. However, the diagnosis of persistent infection at the time of reimplantation is an important part of the diagnostic challenge for patients with failing arthroplasties and we believe inclusion of this subset of patients in the final analysis was instructive.

With expected increases in the aging and obese population, failed hip and knee arthroplasties will remain an important clinical problem. Adverse consequences of misdiagnosis are considerable, with patients being subjected to prolonged antimicrobial therapy and unnecessary surgical procedures. Single clinical and laboratory-based tests cannot reliably diagnose the infected joint, and as we have shown, orthopaedic surgeon’s diagnostic determinations based on a summation and interpretation of many data points had high accuracy. In our continuous quest to improve the standard of care in an era of cost optimization, our audit of orthopaedic surgeon’s diagnostic accuracy is timely. We did not identify any preoperative parameters that outperformed their clinical decision-making, but routine implementation of histopathology could improve physician performance. Importantly, we observed that microbiological results including those generated using advanced molecular methods may confound the clinical decision making process. Given the complexities in interpreting microbiological investigations, we believe that a combination of both culture and molecular methods may be required depending on the clinical context, and their results should be interpreted with caution and tailored to each unique clinical presentation. We hope this study may serve as a guide for future research efforts in exploring the multiple determinants of clinical decision-making for the infected joint in clinical practice, and demonstrates the need for rigorous examination of the added value of tests to improve our effectiveness as clinical decision makers that lead to better patient outcomes.
